# Editorial: Regulation of Adult Stem Cells Fate and Function in Natural and Artificial Microenvironments

**DOI:** 10.3389/fcell.2022.955568

**Published:** 2022-08-17

**Authors:** Pavel I. Makarevich, Yu-Chen Hu

**Affiliations:** ^1^ Laboratory of Gene and Cell Therapy, Institute for Regenerative Medicine, Lomonosov Moscow State University, Moscow, Russia; ^2^ Chair of Biochemistry and Molecular Medicine, Faculty of Medicine, Lomonosov Moscow State University, Moscow, Russia; ^3^ Department of Chemical Engineering, National Tsing Hua University, Hsinchu, Taiwan

**Keywords:** stem cell niche, tissue engineering, extracellular matrix, mesenchymal stem cells, differentiation

## Challenging Nature of Adult Stem Cell Fate Control Mechanism for a Researcher

The stem cells of an adult organism possess an amazing ability to support tissue renewal throughout life and take part in a complex framework of events after damage, where they present a valuable source of tissue “spare” elements. Their shift from quiescence towards differentiation or self-renewal is controlled by a *niche* microenvironment that provides an interface. The latter “filters and amplifies” incoming signals to generate a multifaceted yet unambiguous instruction for residing stem cells. As a theoretical basis, the concept of the stem cell niche is a robust model describing the general principle of stem cell fate control. However, modeling the stem cell fate and its time-course apart from native tissue is complicated. In some cases (stromal niches) even the entity itself is described as a dynamic focus of stimuli and can hardly be localized for investigation.

In this Research Topic, the majority of researchers focus on chosen objects to hypothesize their role in stem cell control and put them to experimental tests to conclude on their importance in cell fate definition. Studied objects in this field range from matrix proteins and cytokines to transcription factors or synthetic biomaterials that mimic the tissue conditions or at least some of them.

## Extracellular Matrix: Role Beyond Structural Support

After a seminal work by Hauschka and Konigsberg (1966), the extracellular matrix (ECM) was considered more than a network of fibrillar proteins providing structural support for cells. The development of decellularization to obtain dECM samples from cultured cells and organs opened a new field extensively studied in recent decades. Investigation of the role of ECM in the control of stem cell differentiation and reprogramming is a growing field and the majority of works in this Research Topic are related to ECM and stem cell fate.

Summary of recent data on ECM in control of cardiac renewal and regeneration has been presented by Pagliarosi et al. from the R. Gaetani group. They present a very original view of ECM as a complex bioactive network that facilitates, regulates, and may control cell fate is presented and supported by strong data including findings by the authors. Stromal/supporting cells, mechanical stimuli, presence of soluble factors, and cellular cross-talk are covered with an accent made to each component’s role in the generation of the microenvironment of the cardiac tissue matrix. Based on these, the authors proposed the application of recent knowledge on the 3D culture and artificial tissues that may reproduce native control of differentiation. A similar level of rigor is found in a review by Ma et al. covering the concept and role of ECM in musculoskeletal plasticity. ECM is pointed to as a critical component of musculoskeletal niches, allowing stem cell entry and exit, transitional niche formation, or modulation of niche composition/environment. This overview of the role of ECM stresses that besides progenitor cell fate, the ECM takes plays an active role in reprogramming mature cells, especially in changing conditions (e.g., damage).

Jiang et al. found that dECM facilitated mesenchymal-to-endothelial phenotype switch in seeded adipose-derived mesenchymal stromal/stem cells (MSC), which correlated with an influx of pro-regenerative M2-macrophages. In contrast, dECM alone was widely invaded by M1-macrophages associated with damage and inflammation. Respectively, MSC-seeded dECM has shown active adipogenesis outcomes in nude mice while dECM alone has failed to provide mature fat at the site of implantation. This is one of few reports on dECM inducing active reprogramming of stromal cells to endothelial ones.

The work of Yang et al. supports the role of ECM in stem cell differentiation control, demonstrating that testis-derived decellularized ECM (dECM) alone was a sufficient platform for mouse spermatogonial stem cell long-term culture and differentiation to spermatids once being induced properly. Besides providing a feasible culture model using dECM hydrogel authors highlight the importance of SSC contact with tissue-specific ECM to function properly. Similar findings using the dECM approach were reported by Novoseletskaya et al., who found that human MSC enhances differentiation potency to 3 lineages (adipose, bone, and cartilage) once cultured on dECM obtained from long-term cultured MSC to obtain cell sheets. These findings suggest the importance of ECM-controlled stiffness and integrin signaling support in the function of stromal-type niches.

Finally, recombinant proteins from non-mammalian species may provide an interesting biomimetic approach to induce and support neural progenitor cells (NPC) *in situ*. Moisenovich et al. used recombinant spidroin 1 (rS 1/9)—an insoluble insect scleroprotein (much resembling animal collagens) to successfully activate nestin-positive NPCs at the site of brain damage in mice. The putative mechanism included activating the influence of rS 1/9 degradation products on resident NPCs. Thus, a xenogeneic protein was successfully used to invoke pro-regenerative and survival effects in mammalian resident NPC.

## Tissue-and Site-Specificity Defining Stem Cells and MSC Properties

A cell’s origin provides a significant bias even when it is isolated and cultured for a significant period of time. Epigenetic and transcriptomic fingerprints defined by tissue and developmental environment still influence how the cell acts and functions *in vitro* making them autonomous properties of these cell cultures. This becomes especially important when we isolate primary MSC or stem cells for the needs of regenerative medicine (cell therapy or tissue engineering). In that case, both efficacy and safety may be impacted by tissue and site of origin.

The autonomous properties of cells driving their regenerative potency and cell fate are the focus of a hypothesis paper by Kulebyakina and Makarevich, who suggests that resident MSC retaining high expression of *Hox* genes after birth might be the “pro-regenerative” subpopulation responding to damage differently from *Hox-*negative MSC. Furthermore, they draw attention to the regenerative capacities of human endometrium and speculate on the role of HOXA10 and HOXA11 in the mediation of numerous healings without scarring that occurs in every menstrual cycle. Thus, a novel role of *Hox* genes (widely known as the cell’s positional “bar-code”) has been proposed in tissue renewal which involves cyclic damage of endometrium.

A good example of site-specificity influence on stem cell fate is provided by Groeneveldt et al. Comparative analysis of skeletal stem and progenitor cells (SSPC) from the periosteum of maxilla, mandible, or tibia demonstrated that despite the large extent of similarity, anatomic origin has a great impact on osteogenic properties of periosteal SSPC. For example, in ectopic bone formation animal test maxilla-derived periosteal SSPC failed to generate bone while cells from the mandible were as effective as tibial ones. This highlighted that even cells of similar tissue origin possess site-dependent features which should be evaluated and taken into account for regenerative medicine and tissue engineering implication of those.

Wang et al. focused on the site-dependent profile of adipose-derived MSC isolated from subcutaneous fat or infrapatellar fat pads trying to imply a concept of tissue-specific lineage preference during differentiation. Using transcriptomic and proteomic analyses they successfully identified a significant discrepancy between these cell types depending on their source. They demonstrated that cells from the subcutaneous compartment showed higher proliferation and an incline towards adipogenic differentiation compared to their infrapatellar fat counterparts that were pro-chondorgenic. One of the putative reasons for autonomous properties might be a significant difference in local ECM environments highlighted in other papers in this Research Topic.

## Development-Driven Approach for Understanding Stem Cell Fate Regulation Mechanisms

Cell fate shifts during the development of an organism are a well-studied field in embryology and is a fruit field for investigation of adult stem cells’ properties and modes of operation under different settings.

Zhang et al. provide a cutting-edge development-driven study, shedding light on the asymmetrical pattern of growth in cartilage formation that eventually allows the formation of sophisticated shapes in these elements of the human skeleton. Lineage tracing experiments of Col2+, Prrx1+, and Gli1+ cells provided an important point that a switch between cell division and cell influx exists immediately after joint cavitation to form epiphyseal cartilage. These findings contribute to understanding of factors that control the shape of organs and tissues and make way to further targeted investigation of mechanisms that govern these cells’ subpopulations and their cross-talk.

A review by Kurenkova et al. from the A Chagin Group focuses on the above-mentioned SSPC, with a focus on their niches in different compartments. A thorough summary of current literature proposes that all described niches have a site-specific repertoire of signals that can be divided into *primary* or *modulating* depending on their role: maintaining basic SSPC functions (renewal or progeny generation) or adaptation to a change of conditions respectively. Eventually, among a myriad of signaling molecules Wnt, hedgehog, and inflammatory cytokines were highlighted as indispensable regulators in all described niches of SSPC of mesenchymal origin.

A review by Milichko and Dyachuk provides insights into the function of glial cells, focusing on the recently discovered heterogeneity of Schwann cells, stemness of certain sub/populations of the human nervous system, and their origin in development. Extensive comparative and descriptive analysis of both embryonic and adult Schwann cells and their potency draws attention to their future use to stimulate nerve system renewal or regeneration.

## Developing Models, Sophisticated Techniques, and 3D Cultures to Control Stem Cell Fate and Viability

Significant attention has also been paid to techniques and models that can be implied for stem cell research, disease modeling, or used to support stem cells’ properties *in vitro.* Yang et al. developed an original model implying a magnetically-driven dynamic system to culture alveolar cells in circulating flow, providing mechanical stimuli for adequate long-term culture and distribution of cells. Furthermore, the authors have demonstrated that silica particles uptake to validate functional properties and obtained a controllable and highly reproducible model that allows investigation of lung physiology and disease in a tissue-like environment*.*

The “Hanging drop” model was developed by Cho et al. using a sophisticated model of this classic approach involving a pressure-assisted network for droplet accumulation (PANDA). The tunable and robust generation of artificial niches was demonstrated and 3D microtissues from MSC and podocytes were obtained to eventually suggest their use in tissue-mimicking with the expansion of PANDA application to other niches and cell types. An approach for cell sheet generation has been proposed by Cheng et al., who used human platelet lysate (HPL) to obtain thicker cell sheets enriched with connective tissue and demonstrated the anti-fibrotic and pro-angiogenic properties of their secretome. The soluble factors of HPL might find use in the generation of tissue-engineered constructs for tissue repair and replacement.

The spatial status of cells and compaction are highlighted by Domnina et al. in their study, which used endometrial MSC in spheroids and found that compaction resulted in cell protection from oxidative stress. Interestingly, compaction decreased anti-apoptotic and anti-autophagy genes suggesting a pathway to programmed elimination of damaged cells in 3D culture. The authors demonstrated a HIF-1α-dependent increase in the therapeutic efficacy of secretome samples obtained from spheroids compared to routine culture. Thus, a switch to a 3D environment altered the MSC viability control program and regenerative capacity. A paper by Hu et al. also focused on cell viability control and showed that apo- and necroptosis in nucleus pulposus-derived stem cells due to compression may be activated via heat shock protein 90 (HSP90). Beyond a basic understanding of a widely spread degenerative disease, this study provided an interesting therapeutic target for future drug development.

## The Twist of Fate: Paving the way to Cellular Reprogramming

Cell fate and control are associated with many amazing phenomena including rapid change of cellular identity from one type to another or induction of pluripotency *in vitro.* For many researchers and translational scientists working in regenerative medicine controlled reprogramming of adult cells it remains a goal to be reached and the current Research Topic covers certain endeavors in the field.

A bioinformatic study by Wang et al. involved a wide array of stem cells from mouse embryo adult testis, brain, and bone marrow using a MEF cell line as control. Their transcriptomic analysis provided an array of genes (*Itgam, Cxcr6,* and *Agtr2*) that might overlap and bridge between cell type-specific regions and thus in the future may provide targets for reprogramming control or stimulation of SC renewal. Seeking molecular pathways and targets that may induce cell transdifferentiation has led Dai et al. to a study where canine adipose MSC and insulin-producing cells (IPC) were screened, and eventually *Foxa1, Pbx1* and *Rfx3* were validated to provide prospective targets for induction of MSC-to-IPC reprogramming in future experimental protocols and cell-replacement therapy.

Among soluble factors that regulate cell fate extracellular vesicles (EVs) were in the spotlight and our Research Topic included a work by Lopatina et al. who convincingly demonstrated that EVs released from tumor endothelial cells are mediators of non-metastatic carcinogenesis. A potent ability of tumor-derived EVs to “corrupt” local immunity via reprogramming of stromal and immune cells by factors carried in tumor-generated EVs have been shown.

## Concluding Remarks

The articles collected in this Research Topic indicate that contemporary understating of how a cell “commits and performs” implies the existence of a complicated network of signals, stimuli, and interactions that guide towards maturation, putting it to a halt or even reverse. Researchers investigating how stem cell fate is defined will endeavor to evaluate each known element’s role and impact. Futures challenges will soon need to be addressed, such as when we try to understand how the integral complex system of cell fate definition works.

Despite these challenges, we believe that a precise description of the crucial factors that control and comprise the niche and provide tissue-specific features accompanied by the development of new techniques to investigate cell fate will eventually build a new understanding of how stem cells are controlled in different settings.

## References

[B1] Hauschka S. D. Konigsberg I. R. (1966). The Influence of Collagen on the Development of Muscle Clones. Proc. Natl. Acad. Sci. U. S. A. 55 (1), 119–126. 10.1073/pnas.55.1.119 5220860 PMC285764

